# Sudden Cardiac Death: A Systematic Review

**DOI:** 10.7759/cureus.42859

**Published:** 2023-08-02

**Authors:** Arturo P Jaramillo, Mohamed Yasir, Nandhini Iyer, Sally Hussein, Vijay Prabhu SN

**Affiliations:** 1 General Practice, Universidad Estatal de Guayaquil, Machala, ECU; 2 Internal Medicine, Kursk State Medical University, Kursk, RUS; 3 Research, California Institute of Behavioral Neurosciences & Psychology, Fairfield, USA; 4 Internal Medicine, Grant Government Medical College and Sir JJ Group of Hospitals, Mumbai, IND; 5 Internal Medicine, California Institute of Behavioral Neurosciences & Psychology, Fairfield, USA; 6 Internal Medicine, Vardhman Mahavir Medical College (VMMC) and Safdarjung Hospital, New Delhi, IND

**Keywords:** cardiac arrest outcome, implantable-cardioverter defibrillator, obstructive sleep apnea (osa), phase-i cardiac rehabilitation, sudden cardiac death

## Abstract

Sudden cardiac death (SCD) is a condition that accounts for a high percentage of cardiovascular fatalities, with ventricular tachyarrhythmias being the most common cause. There are signs and symptoms of SCD that occur spontaneously without any warning and are deadly. Despite preventative efforts focusing on the use of subcutaneous implanted cardioverter defibrillators (S-ICD) in the highest-risk population categories, a high number of SCDs occur in the normal population and in people who do not have a documented cardiac condition. Therefore, primary prevention for SCD should be a more viable strategy for the general population, considering measures in the form of preventive medicine such as knowing more about any genetic predisposition, family history of any fatal arrhythmia, continuous surveillance after any syncope with unknown causes, etc. However, little data about SCD risk factors are known in comparison with other well-known diseases like ischemic heart disease and stroke. In search of medical databases for relevant medical literature, we looked at PubMed/Medline, the Cochrane Library, and Google Scholar. Fourteen publications were discovered after the papers were located, assessed, and qualifying criteria were applied. The finished articles were done to give an overview of SCD. Some others have shown that the major predisposition for SCD is related to the male gender, which increases the incidence if they have a family history of SCD. We described the importance of obstructive sleep apnea (OSA) as a comorbid condition. Patients with S-ICD and young athletes with a history of ventricular arrhythmia showed us that the predisposition for SCD can be higher than in the normal population. Based on the above, we concluded that more study is required to establish the most important approach for each of the risk factors mentioned in this systematic review in order to apply them in daily practice and have more knowledge about how to apply preventive and therapeutic medicine to the population at risk and the ones that already develop the disease.

## Introduction and background

Sudden cardiac death (SCD) is one of the top causes of death in the United States, claiming the lives of 300,000 to 350,000 people each year [1]. Cardiovascular disorders like coronary artery disease (CAD), intrinsic electrical abnormalities, congenital heart disease, and cardiac muscle pathology are a combination of underlying disease pathologies that contribute to SCD. Men are more likely than women to have SCD, and the underlying heart disease varies across the sexes. CAD is more common in men than in women, making it the most frequent underlying cause of SCD [2,3]. In women with SCD, nonischemic pathology, such as myocardial fibrosis, arrhythmogenic right ventricular cardiomyopathy, and valvular heart disease, is more frequent than in males with the same affectation. Women are less likely than men to have a history of heart disease, and SCD is the initial indication of heart disease in this population [3,4]. In women, SCD has a different presentation, maybe due to variances in the illness, resulting in a risk factor disparity.

Another risk factor for SCD is having QT disorder, or short QT syndrome, which is a sign of ventricular abnormality. As a general fact, it is mentioned that once puberty is attained, women have longer QT intervals than men. Furthermore, prior research showed that the increased risk of total cardiac mortality linked with a longer QT interval was greater in women. If a woman has an inherited or acquired QT prolongation, she is more likely to have arrhythmic episodes compared to a man. Similarly, it is thought that QT interval may alter SCD differently depending on a case-by-case basis (e.g., ischemic vs. nonischemic illness) [1,2]. The most effective prevention treatment used so far for these fatal cardiac arrhythmias is the S-ICD, which is now implanted more often in bigger tertiary referral institutions than in smaller facilities around the US. It has been seen in several observational studies that, with time, the rates of complications decreased [5]. People who have survived SCD or who have had an S-ICD come from a wide range of preexisting health conditions and chronic heart disease. As a result of resuscitation, SCD survivors have specific cognitive, psychological, emotional, and functional demands [3]. The quality of life suffers when people with these special requirements are unable to go back to work, resume their regular routines, or fulfill their social and familial responsibilities. Surviving a sudden cardiac arrest (SCA) may have long-term repercussions, according to the available data [3].

Different methods have been proposed for locating patients who have hypertrophic cardiomyopathy. Several clinical studies have looked at traditional risk factors, such as a history of the disease in the family, non-sustained ventricular tachycardia on 24-hour Holter monitoring, severe ventricular hypertrophy, SCD, syncope that could not be explained, and an abnormal response of blood pressure to exercise. As a result, the pathogenesis of SCD in hypertrophic cardiomyopathy is still poorly understood. Several hypotheses, including the progression of cardiac fibrosis, seem to be implicated in this issue [6,7]. In addition, those who have end-stage renal disease and are in need of therapy that replaces their kidney function (dialysis) have a mortality risk that is 14 times higher than people who do not have cardiovascular disease. Cardiovascular illness is the most common cause of death in these patients. Different treatments, especially with S-ICD, have been beneficial in lowering SCD mortality. Arrhythmias and cardiac arrest (CA) are thought to account for 30% of all deaths. Accordingly, the S-ICD was created to identify and treat potentially fatal ventricular arrhythmias using defibrillation, cardioversion, or antitachycardia pacing [8,9].

This systematic review aims to provide an overview of SCD, with a particular focus on the illness's risk factors, pathophysiology, and associated comorbidities. The overarching goal is to obtain a deeper understanding of this uncommon condition that claims a significant number of lives each year. In addition, we provide a brief overview of the value of S-ICD therapy as well as the rates of efficacy in a variety of illness associations that have led to the development of this condition.

## Review

Methodology

We performed a systematic evaluation using free full-length papers and the Preferred Reporting Items for Systematic Reviews and Meta-Analyses (PRISMA) checklist to describe our approach and results. 

Study duration

This review started on June 1, 2023.

Search strategy

PubMed, Google Scholar, and Cochrane were used to collect the database using the following: ("Death, Sudden, Cardiac/etiology"[Majr:NoExp] OR "Death, Sudden, Cardiac/pathology"[Majr: NoExp] OR "Death, Sudden, Cardiac/prevention and control"[Majr:NoExp] ), AND ( "Heart Arrest, Induced/education"[Majr:NoExp] OR "Heart Arrest, Induced/mortality"[Majr:NoExp] OR "Heart Arrest, Induced/rehabilitation"[Majr:NoExp] OR "Heart Arrest, Induced/trends"[Majr:NoExp] ), AND ( "Defibrillators, Implantable/adverse effects"[Majr:NoExp] OR "Defibrillators, Implantable/ethics"[Majr:NoExp] OR "Defibrillators, Implantable/standards"[Majr:NoExp] OR "Defibrillators, Implantable/trends"[Majr:NoExp] ) 

Eligibility criteria and study selection

To assess eligibility, two investigators carefully read the full title and content of each paper. We selected the latest literature and the articles published in the past five years, including papers written in the English language, or if the full free-text English-language translation is available. Articles were excluded if the full text of the papers could not be retrieved. Articles focusing on SCD were strictly chosen. Gray literature and proposal papers were also not included.

Data management

Two independent writers evaluated papers based on titles and abstracts. Following that, significant abstracts were examined for a complete, free full-text examination. The selected studies were evaluated, and if there was any dispute, the research was evaluated by a third author. Information from relevant publications was then collected. The first author's name, type, year of publication, study design, and results were taken as priorities. Finally, duplicates were deleted.

Quality assessment

We used the Assessment of Multiple Systematic Reviews (AMSTAR) form and the Cochrane risk-of-bias assessment tools for clinical trials and for systematic reviews and meta-analyses. 

Results

Search Results

**Figure 1:**
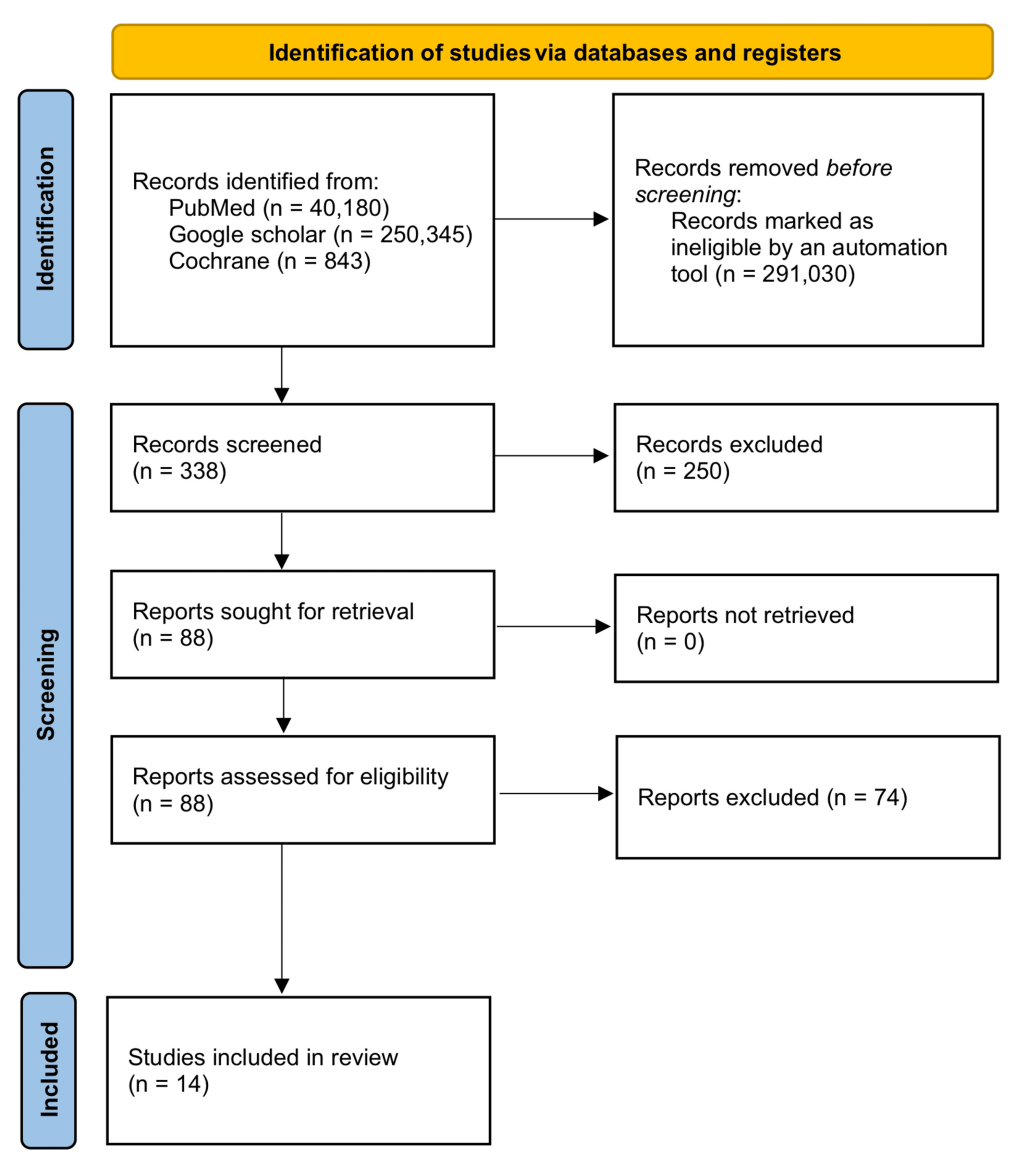
Identification of studies via databases and registers

A total of 291,368 studies were found after searching PubMed, Google Scholar, and the Cochrane Library. A total of 291,030 studies were marked as ineligible by an automation tool. There were a total of 338 studies that underwent title and abstract screening, with 250 papers being discarded. The remaining 88 papers were chosen by full-free text evaluation in the previous two years, and after discarding duplicates, resulting in the elimination of 74 studies, only 14 studies were enlisted for the final collection of data (Figure 1).

Table 1 shows a summary and characteristics of all included studies.

**Table 1:** Table of data extraction LVEF: left ventricular ejection fraction; SCD: sudden cardiac death; S-ICD: implantable cardioverter defibrillators;  SCT: social cognitive theory; HR: heart rate; RCT: randomized controlled trial.

Author	Year of publication	Study design	Quality tool	Primary research	Outcome evaluation
Eiger et al. [2]	2022	RCT	Cochrane risk-of-bias assessment tool	2521 patients with heart failure classes II and III with ischemic or non-ischemic cardiomyopathy and an LVEF of 35% were randomly given, amiodarone, placebo, or S-ICD shock-only.	LVEF increased significantly in one-third of the patients in this study during subsequent visits; LVEF improvement was related to better survival.
Dougherty et al. [3]	2022	RCT	Cochrane risk-of-bias assessment tool	There were 168 participants in the trial, 129 men and 39 women, who received an S-ICD for the purpose of prevention. These participants were randomly assigned to one of two research conditions: usual care or SCT intervention.	In spite of having participated in an SCT, SCD survivors revealed poorer levels of psychological well-being and physical symptoms after a year, in addition to having lower expectations for the results.
Kurl et al. [1]	2021	RCT	Cochrane risk-of-bias assessment tool	We examined the relationship between slow HR reserve, sluggish HR recovery, and the likelihood of SCD. 1967 males aged 42–61 years were chosen at random.	209 SCD occurrences occurred in 25 years. The SCD rate was elevated due to a worsening heart rate with time.
Heilbrunn et al. [4]	2021	SRL and Meta-analysis	AMSTAR checklist	The databases, Scopus, Cochrane Library, the Joanna Briggs Institute for Evidence-Based Practice, and MEDLINE, were searched from their establishment until January 1, 2020.	There was a correlation between obstructive sleep apnea and SCD from any cause (relative risk = 1.74), as well as cardiovascular mortality (relative risk = 1.94).
Paratz et al. [5]	2020	SRL	AMSTAR checklist	Existing registry resource, highlighting spatial unfairness, and comparing worldwide metrics of SCD detection and adjudication.	There are several cardiac arrest registries across the world, but their population coverage is uneven. SCD registries with comprehensive multi-source monitoring are fewer in number and more difficult to build and manage.
Pan et al. [6]	2020	SRL and Meta-analysis	AMSTAR checklist	2939 SCDs among 418,235 patients were chosen for the meta-analysis from 18 studies.	An elevated risk of SCD in association with a hypertension diagnosis and rising systolic blood pressure was shown.
Furtado et al. [7]	2020	RCT	Cochrane risk-of-bias assessment tool	Ticagrelor vs. placebo for cardiovascular event prevention in heart attack survivors: 54 studies were taken for their respective analyses.	In individuals on ticagrelor after myocardial infarction, consuming caffeine did not reduce dyspnea compared to not consuming caffeine.
Couper et al. [8]	2020	SRL	AMSTAR checklist	Database searches of EMBASE, the Cochrane Library, and MEDLINE were conducted, as well as citation tracking of eligible studies. To be analyzed for a better approach to the study.	The incidence of SCD mortality in the general population was 1.7 per 100 000 person-years (1.3–2.6, range 0.75–11.9).
Aune et al. [9]	2020	SRL	AMSTAR checklist	From conception until March 26th, 2019, Embase and PubMed databases were searched for research on SCD and physical activity.	A high level of physical exercise, as opposed to a low one, may minimize the risk of SCD in the general population.
Nielsen et al. [10]	2019	SRL	AMSTAR checklist	Embase, MEDLINE, CENTRAL, and four additional databases were used, as were three additional trial registrations. In the study, references were also checked for citations.	All-cause mortality was recorded in seven studies, with no indication of a difference between cardiac rehabilitation and the control group, with a risk ratio of 1.96 and a 95% confidence range of 0.18 to 21.26.
Mitchell et al. [11]	2019	RCT	Cochrane risk-of-bias assessment tool	2282 patients with SCD and 3561 controls were selected. Two groups were built based on the underlying conditions of the participants (ischemic vs. nonischemic) and gender.	In the end, we found a strong genetic link between SCD risk patients with nonischemic SCD and the QT interval length, as well as a link that could be a cause.
Kamp et al. [12]	2019	RCT	Cochrane risk-of-bias assessment tool	Observational studies were chosen to investigate if S-ICDs are both effective and safe.	Studies using observational methods have shown that the S-ICD has the potential to be both useful and safe.
Bittencourt et al. [13]	2019	SRL and Meta-analysis	AMSTAR checklist	21 studies were chosen (males made up 62.8% of the sample, with 14,901 patients aged 16–45 years).	Strong arrhythmic outcomes are linked to risk factors, cardiac fibrosis, and hypertrophic cardiomyopathy. This shows how important it is to treat these people with better prediction models.
Wouter et al. [14]	2019	RCT	Cochrane risk-of-bias assessment tool	The S-ICD trial was randomized and controlled and examined the efficacy of the S-ICD in preventing SCD in a group of 200 dialysis patients who had a left ventricular ejection fraction of 35%. The participants were all receiving dialysis.	Treatment with S-ICDs did not reduce the risk of SCD or overall mortality in a well-monitored dialysis population.

Discussion

In this systematic review, we want to emphasize the significance of having an entire perspective on SCD in patients who are totally normal, the ones that have family history, and those who have comorbidities associated with the disease, as well as the pharmacological therapy to prevent it without reverting to the ultimate treatment, which is the S-ICD. A randomized controlled trial (RCT) conducted by Eiger et al. showed that one-third of the patients included in the research showed > 10% improvement in left ventricular ejection fraction (LVEF), demonstrating that the use of S-ICDs is substantially linked with increased survival regardless of the change in LVEF and other clinical characteristics (HR 0.62). This enhancement is more likely to occur in White females that have a QRS duration of 120 milliseconds or less, have a history of hypertension, and/or use beta-blockers Furthermore, there was a decreased likelihood of having appropriate S-ICD therapy and a decreased risk of death from any cause linked with a 10% rise in LVEF [1]. Dougherty et al.'s RCT found a statistically significant indication per time interaction (p = 0.04) for patient concerns and evaluation symptoms of ventricular arrhythmias, indicating that over one year, the changes were lower in SCD survivors vs. other arrhythmias. The quantity of S-ICD shocks differed significantly depending on intervention and S-ICD indication (p = 0.007 for a two-way interaction). Compared to people who did not experience SCD but had an S-ICD implanted for ventricular arrhythmia, SCD survivors who received an initial S-ICD had different recovery trajectories in psychological adjustment, physical function, and self-efficacy outcomes over one year while participating in a social cognitive theory intervention [2,15]. In another study done by Kurl et al., two measures of chronotropic impairment and autonomic nervous system function during exercise testing-delayed heart rate reserve and slow heart rate recovery-were shown to be related to an increased risk of SCD in the studied population. Furthermore, both tardy rate reserve and slow rate recovery after the maximal symptom-limited exertion test predicted the incidence of SCD in men, independent of established cardiovascular disease risk markers [3].

Heilbrunn et al. conducted an RCT and discovered that obstructive sleep apnea (OSA) increases the risk of SCD mortality by twofold [4]. The Fonseca et al. study, which comprised 13,394 individuals from 13 research publications published between 2002 and 2014, was comparable regarding the high risk of mortality that OSA can cause for the development of a spontaneous fatal arrhythmia [4,16]. This link may be explained by the neurological system's effect on the human sleep cycle. OSA causes occasional oxygen deficiency, and hypoxia during sleep, driving the central nervous system to over-arouse to enhance airflow. During apneas, the interaction between the autonomic and sympathetic nervous systems generates an occasional rise in blood pressure [4,17]. According to Paratz et al.'s RCT, the following number of patients were identified: 15 SCD, 49 CAs, and 9 additional registries were gathered during the emergency hospital shift. The population coverage of modern SCD and CA registries varies greatly in comparison to those in the US and Western Europe. According to the aforementioned, the existing SCD registries include a wide range of age subpopulations and groups, with some including family members and surviving patients [5,18].

To the best of our knowledge, Pan et al. conducted a systematic review and meta-analysis on hypertension, blood pressure, and the risk of SCD. Their findings revealed that people with high blood pressure had a doubling in their chance of SCD and a 28% increase in their risk of SCD for every 20 mmHg increase in systolic blood pressure (SBP). However, no significant link between SCD and diastolic blood pressure (DBP) was found. While the results for SCD and hypertension were solid, the analysis for SCD and DBP was limited by the small number of studies included in the review. Nonetheless, a pooled analysis of 61 trials (involving 553 SCD cases and 1 million individuals) partly supported these findings [6,19].

Another study by Furtado et al. investigated the relationship between caffeine consumption and dyspnea in high-risk individuals with a history of myocardial infarction. Their research demonstrated that individuals with a history of myocardial infarction often consume caffeinated beverages. However, consuming coffee did not reduce the prevalence of dyspnea in these individuals. Furthermore, there was no evidence linking caffeine use to an increased risk of clinically significant arrhythmias or ischemic cardiovascular events among people with a history of myocardial infarction. The study compared placebo and ticagrelor doses, and the risk of dyspnea was assessed across different coffee intake categories. Overall, the analysis of data from the Prevention of Cardiovascular Events in Patients With a Prior Heart Attack Using Ticagrelor Compared to Placebo on a Background of Aspirin-Thrombolysis in Myocardial Infarction 54 trial found no evidence that caffeine consumption altered the risk of dyspnea with either a high or low ticagrelor dose [7,20].

In Couper et al.'s analysis, where they reviewed 38 articles reporting the prevalence of SCD in young people, they discovered substantial variation in the reported incidence between trials. The reported incidence ranged from 0.75 to 11.9 cases per 100,000 person-years, with the vast majority of studies reporting an incidence of 1-2 cases of SCD per 100,000 person-years. Such variation might be attributed to changes in demographics, case-ascertainment methodologies, and the definition of SCD used. One important finding was that rising age and male sex seem to be related, with a greater incidence in selected subgroups [8].

According to the research by Aune et al., which included 136,298 individuals with SCD and physical activity, the summary relative risk (RR) for the highest vs. lowest level of physical activity was 0.52, resulting in statistical significance. The connection between physical activity and SCD risk was comparable in men and women, as well as in European and American studies. The dose-response study indicated an RR of 0.68 per 20 metabolic equivalent hours per week. Additionally, the overall RR for the highest vs. lowest level of cardiorespiratory fitness was 0.58, which was statistically significant [9,21].

Nielsen et al. conducted a comprehensive analysis and found eight randomized controlled studies with a total of 1730 individuals who had an S-ICD with or without cardiac resynchronization treatment function and were comparing no exercise control vs. exercise-based cardiac rehabilitation. The analyses revealed that, concerning the endpoint of exercise capability, there was merely an indication of a difference favoring exercise-based cardiac rehabilitation. There was no indication of a long-term benefit from exercise-based cardiac rehabilitation compared to a control group; however, there was some evidence of a short-term benefit. The hypothesis would be that exercise-based cardiac rehabilitation had no long-term effect on exercise behavior in the included subjects and thus had no postponed benefit [10,22].

## Conclusions

This systematic review aimed to provide an overall understanding of SCD in patients who are completely normal, those with a family history of the disease, and those with comorbidities related to SCD, as well as to explore pharmacological therapies for prevention, without solely relying on the ultimate treatment, which is the S-ICD device. Based on the articles reviewed, patients who underwent S-ICD showed a better outcome related to LVEF, with variations seen in different associations like gender, race, and ECG morphology.

Moreover, the review highlighted a greater susceptibility of men to SCD, which was associated with a low cardiac rate during exercise. Another significant comorbidity related to SCD is OSA, especially when accompanied by other conditions like hypertension, obesity, or type 2 diabetes mellitus. Consequently, these factors may have a real effect on SBP and DBP changes in the heart, increasing the risk of potentially deadly arrhythmias.

In light of the above, it can be concluded that SCD can be caused by various comorbidities, including male gender, increasing age, family history of SCD, use of drugs that can prolong the QT interval, OSA, and undiagnosed congenital heart defects. Therefore, more large-scale studies are needed to assess which of these comorbidities presents the greatest risk of generating SCD, and to understand the importance of establishing different approaches for prevention (such as lifestyle modification) and treatment (including drug therapy) without solely relying on the definitive treatment of S-ICD.
