# Correction: Sudden Cardiac Death: A Systematic Review

**DOI:** 10.7759/cureus.c271

**Published:** 2025-08-26

**Authors:** Arturo P Jaramillo, Mohamed Yasir, Nandhini Iyer, Sally Hussein, Vijay Prabhu SN

**Affiliations:** 1 General Practice, Universidad Estatal de Guayaquil, Machala, ECU; 2 Internal Medicine, Kursk State Medical University, Kursk, RUS; 3 Research, California Institute of Behavioral Neurosciences & Psychology, Fairfield, USA; 4 Internal Medicine, Grant Government Medical College and Sir JJ Group of Hospitals, Mumbai, IND; 5 Internal Medicine, California Institute of Behavioral Neurosciences & Psychology, Fairfield, USA; 6 Internal Medicine, Vardhman Mahavir Medical College (VMMC) and Safdarjung Hospital, New Delhi, IND

This article has been corrected to fix the following typos present in the Abstract, Search Results section, and PRISMA diagram:

- Abstract: “**Thirteen** publications were discovered after…” has been corrected to "**Fourteen** publications were discovered after..."
- Search Results: “There were a total of 338 studies that underwent title and abstract screening, with **257** papers being discarded. The remaining 88 papers were chosen by full-free text evaluation in the previous two years, and after discarding duplicates, resulting in the elimination of **75 **studies, only **13** studies were enlisted for the final collection of data (Figure 1).” has been corrected to “There were a total of 338 studies that underwent title and abstract screening, with **250** papers being discarded. The remaining 88 papers were chosen by full-free text evaluation in the previous two years, and after discarding duplicates, resulting in the elimination of **74** studies, only **14** studies were enlisted for the final collection of data (Figure 1).”
- PRISMA diagram:
  - "Records excluded (n=**257**)" corrected to "Records excluded (n=**250**)"
  - "Reports excluded (n=**75**)" corrected to "Reports excluded (n=**74**)"
  - "Studies included in review (n=**13**)" has been corrected to "Studies included in review (n=**14**)"

